# Maintenance of ophthalmic specialist out-patient service during the COVID-19 outbreak: The University of Hong Kong experience

**DOI:** 10.1038/s41433-020-0887-z

**Published:** 2020-04-21

**Authors:** C. Kendrick Shih, Jonathan C. H. Chan, Jimmy S. M. Lai

**Affiliations:** Department of Ophthalmology, The University of Hong Kong, Hong Kong, Hong Kong

**Keywords:** Scientific community, Health care

## To the Editor:

The coronavirus disease 2019 (COVID-19) has led to more than 0.3 million confirmed cases and 13,581 deaths worldwide. As of today, there are 317 confirmed cases in Hong Kong. We share our local experience in the provision of ophthalmic out-patient service during the current COVID-19 outbreak. COVID-19 is highly contagious, with evidence of viral shedding in tears and ocular secretions of affected individuals [1]. The American Academy of Ophthalmology released recommendations on the on March 18, 2020, that all ophthalmologists should cease providing treatment other than urgent care in clinics and hospitals [2]. The recommendation would result in significant disruption to the continuity of ophthalmic care for large number of patients.

To maintain service and minimize disturbance to patients’ management, while ensuring safety to medical staff and patients, a series of infection control measures were adopted at the Lo Fong Shiu Po Eye Centre, Grantham Hospital, after the Hospital Authority of Hong Kong announced activation of emergency response level on January 25, 2020. These control measures, involve firstly, stationing two staff at the reception area to screen patients for fever (through body temperature measurement), respiratory symptoms, and recent travel history to outbreak areas. The electronic medical record system at the clinic was linked to the Immigration Department of Hong Kong, for confirmation of patients’ travel history. Patients with any positive finding had their appointment postponed. Patients arriving without surgical masks were provided one. Secondly, all clinic staff were supplied with surgical masks, appropriate personal protective equipment (PPE), and alcohol disinfectant. Designated rooms were available for donning of protective gowns. Barrier plastic shields were installed on slit lamp to prevent droplet transmission of COVID-19. Thirdly, all clinic procedures that might generate micro-aerosol such as noncontact tonometry [3], or involve tear, fluid or blood spilling, such as nasolacrimal duct syringing and incision/curettage of chalazion were avoided. Instilling eye drops and rinsing fluorescein stain from ocular surface could theoretically cause splashes. Nursing staff performing these procedures were equipped with eye shields. Botox injection for lid diseases were postponed because patients’ masks had to be removed during the injection. Intravitreal injections and various ophthalmic laser treatments were continued for scheduled patients.

Between January 29 and March 21, 2020, the total number of out-patient clinic attendance was 8254, intravitreal injection 348 and ophthalmic laser treatment 191. A 100% compliance rate to wearing surgical masks was observed among clinical staff and patients. There was no reported COVID-19 infection in any of the clinical staff or patients who attended our clinic in this period. While the out-patient service was scaled down to minimize patient flow, if strict adherence to the infection control measures described are taken, it may be safe to maintain essential out-patient clinical services such as ophthalmic consultations, laser treatments and intravitreal injections during the COVID-19 pandemic.
